# Predictors of intention to stay in the profession among novice nurses: a cross-sectional study

**DOI:** 10.1186/s13584-024-00662-4

**Published:** 2024-12-23

**Authors:** Bella Savitsky, Rachel Shvartsur, Ilya Kagan

**Affiliations:** Department of Nursing, School of Health Sciences, Ashkelon Academic College, Yitshak Ben Zvi 12, Ashkelon, Israel

**Keywords:** Intention to stay, Retention, Novice nurses, Job satisfaction, Workplace environment

## Abstract

**Background:**

Preserving new graduate nurses in the profession is an essential step for addressing the nursing shortage and sustaining the future of the profession. This study aimed to examine the relationship between employment characteristics and job satisfaction of novice nurses and their willingness to stay in the nursing profession in the next 5 years.

**Methods:**

Novice nurses’ intention to stay in the profession was assessed, considering demographics, employment characteristics, and components of job satisfaction. Among the sample of 216 novice nurses (93% response rate), four components of job satisfaction were extracted and included in the multivariable logistic regression model with the intention to stay in the profession as a dependent variable.

**Results:**

Professional self-accomplishment was significantly and positively associated with the intention to stay in the profession, with an elevation of one standard deviation in this component associated with more than a two-fold increase in the odds of staying (OR = 2.3, 95% CI 1.3–3.9). This component contributed 10% to the variance in intention to stay. Independently, managerial support also contributed 10% to the variance and was significantly associated with willingness to stay (OR = 1.9, 95% CI 1.2–3.0). Overall, self-accomplishment, managerial support, and healthier organizational culture were significantly associated with novice nurses’ intention to stay, whereas work conditions and rewards were not. The multivariable analysis model explained 38.0% of the variance in the intention to stay in the profession.

**Conclusions:**

This study found that novice nurses’ intention to stay in the profession is highly associated with their self-accomplishment and better managerial support. Thus, to enhance the retention of novice nurses, managers must establish an environment that fosters professional development and support. This involves providing engaging work assignments, facilitating the seamless integration of novice nurses into the team, and offering managerial support and guidance.

## Introduction

According to the World Health Organization (WHO), there was a global nurse shortage of almost six million nurses in early 2020 [1]. The COVID-19 pandemic has exacerbated the immediate need for nurses in all countries. Moreover, this situation has contributed to burnout, significantly threatening nurses’ retention [2–6], resulting in a future need for 13 million more nurses by 2030 [7]. Likewise, Israel is struggling with a shortage of nurses, with a ratio of 6.5 nurses per 1000 residents [8, 9], contrasting with an OECD country average of nearly nine nurses per 1000 residents [10].

To address the shortage, governments are working to increase the number of nursing graduates [8, 12–15]. Unfortunately, while nursing schools spend time and effort training new nurses, many novice nurses leave the profession within the first years after graduation [9, 11–13]. During the COVID-19 pandemic, registered nurses with a decade or fewer years of experience left the profession at an accelerated rate, constituting nearly 41% of the overall decline in practicing registered nurses [13]. Another 15.2% reported plans to leave nursing within the next 5 years [12]. The situation is even more challenging among newly graduated nurses in their first year [9, 14]. Novice nurses experience a transition period characterized by increased stress, anxiety, and worries [15]. Heavy workloads, staff shortages, bullying, limited experience, and high expectations from colleagues render this period crucial, significantly influencing new nurses’ decision not only to stay or leave the organization but also to continue in or exit the nursing profession [16, 17]. Therefore, to avert shortages, there is a need to increase the number of nursing graduates and retain these graduates in the health system [1, 18].

Nurse turnover can be categorized into two types: organizational turnover, where nurses leave their current employer but remain in nursing, and professional turnover, which involves exiting the nursing profession altogether [19]. This distinction is crucial for understanding the different factors influencing novice nurses’ decisions to stay or leave, as the underlying stressors for these two types of turnover often differ. Studies show that organizational turnover is associated with immediate work-related stressors such as heavy workloads, lack of control over schedules, and unsupportive management [14, 20]. In contrast, professional turnover is driven by intrinsic factors, such as emotional exhaustion and a lack of fulfillment in the profession [21]. Notably, organizational turnover is more prevalent among novice nurses than professional turnover; for example, according to the 2022 Nursing Solutions report [22], a third of novice nurses leave their jobs within the first year of employment, though many remain in the profession [22]. While professional turnover directly contributes to the national nursing shortage by removing a full-time equivalent (FTE) or part of an FTE from the workforce, organizational turnover can also have an impact. Gaps in employment between leaving one institution and starting at another can temporarily reduce the available workforce. Additionally, if a nurse transitions from a full-time position at one organization to a part-time position at another, this shift effectively reduces the overall FTEs available. These dynamics highlight the importance of addressing both organizational and professional turnover to mitigate the nursing shortage.

**Fig. 1:**
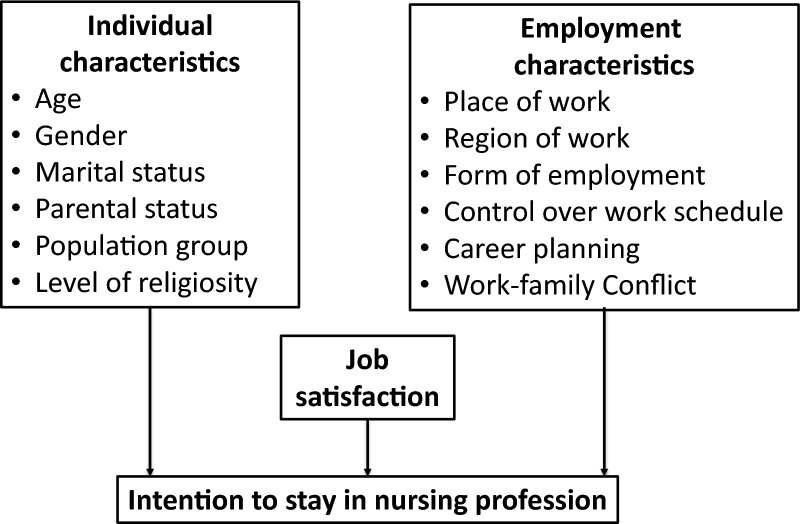
Conceptual framework of the study. Conceptual framework illustrating the predictors of newly graduated nurses’ intention to stay in the nursing profession

Despite numerous studies on nurse organizational turnover, understanding the factors explaining the phenomenon of leaving the profession, especially among new graduates, is still limited [19]. Based on previous studies [19, 23, 24], a few features of newly graduated nurses’ professional turnover intention have been examined in this study. The predictors were classified into individual characteristics, employment characteristics, and job satisfaction (Fig. 1).

*Individual characteristics* (age, sex, marital and parental status, population group, and level of religiosity) have been shown to be associated with professional turnover/retention intentions [24–27]. Higher turnover rates were found among young nurses [9, 28–30]. A study based on the data on all nurses in Israel [9] showed a 77% likelihood of young nurses remaining in the profession after 10 years versus 94% and 99% for older age groups.

Studies report controversial results regarding sex differences in turnover. While some studies found that being male is associated with shorter tenure in nursing [28, 30, 31], others have reported no sex differences [32]. Economic and family status independently affect professional turnover. The likelihood of leaving work as a nurse was significantly lower among RNs with no children under 18 [9, 30], minorities, or immigrants [28].

*Employment characteristics* that were found to be associated with RN retention are place of work (hospital vs. community), form of employment (full vs. part-time), level of control over scheduling, and career planning. Working part-time [14, 28] and having greater control of scheduling [33] were associated with decreased turnover intentions. On the other hand, a low perception of career advancement opportunities was associated with increased intent to leave the profession [29, 30]. A study from Israel revealed that expectation for promotion was associated with nurses’ retention [9]. We chose to address career and educational planning as an employment characteristic because RNs in Israel need approval from their nurse manager to start post-basic studies and receive organizational fiscal support.

Research on retention and turnover of nurses has also emphasized the importance of work-family/home balance and job satisfaction. The need to combine work and family demands leads to work-family conflicts, which predict organizational and professional turnover [19, 23, 24]. A study of 28,561 hospital nurses from ten European countries found that a high work-family conflict was associated with a twofold increased risk of intent to leave the profession [30]. Higher *job satisfaction* was consistently found to be a significant predictor of professional retention [19, 24, 34–37]. Our previous study was dedicated to understanding the components of job satisfaction among novice nurses [38], and these components were used in the current study as predictors of willingness to stay in the profession.

## Objectives

The study aimed to examine the relationships between individual and employment characteristics, job satisfaction, and intention to stay in the nursing profession among novice nurses.

## Methods

### Study population and data collection

We reached out to all graduates (n = 252) who completed a 4-year academic RN program in our college between 2018 and 2022, contacting them via email at least 10 months post-graduation to invite their participation in an online questionnaire. 234 novice nurses completed the survey (93% response rate). Graduates who indicated that they did not commence employment as nurses after graduation were excluded from the study. Consequently, the study encompassed a total of 216 newly qualified nurses.

### Ethical considerations

Each participant provided a signed informed consent to participate in the study, and the research obtained approval from the Ethical Board of the Department of Nursing on November 11, 2020.

### Tools

The survey instrument included the following demographic variables:

Age: Treated as a continuous variable; Sex: Female or male; Family status: Married/in a relationship or single/divorced; Parental status: Has children/does not have children;

Country of birth: Israel/other countries; Ethnicity: Jewish/Arab/Bedouin; Level of religiosity: secular (nonobservant)/traditional (observes some religious commandments)/religious (observes all religious commandments).

Additionally, the survey included information on the professional variables:

Place of work: hospital/community; Region of work: South/Center/North/Jerusalem and environs); Form of Employment: full-time job/part-time job; Level of control over work schedule: low/medium/high); Frequency of work disturbances to everyday life: never/sometimes/always; Advanced course: planning to participate or already participating /not in the plan); Master’s degree: planning to study or already studying/not in the plan.

The survey included a questionnaire designed to assess professional satisfaction, comprising 26 items related to professional satisfaction. Twenty-two items were derived from the Minnesota Satisfaction Questionnaire (MSQ) (short version translated to Hebrew [39]), while four additional items were included at the request of three senior nurses working in a general hospital. The reliability of the MSQ questionnaire, as measured by Cronbach`s Alpha in Israeli studies, was reported as 0.95 [40] and 0.89 [41]. Items were rated on a 5-point Likert-type scale (1 = to a very small degree to 5 = to a very high degree). The reliability of this tool in this study was high (Cronbach’s Alpha method = 0.94) [38].

Job satisfaction components were constructed using factor analysis [38]: the factor of self-accomplishment, related to feelings of worthwhile accomplishment, a challenge at work, the extent of diversity and interest in the professional duties, the use of skills, personal growth and development, contribution to the patient’s care, prospect for promotion, and the extent of independency in the job; the factor of organizational culture, related to satisfaction with relationships with other nurses, the amount of support from coworkers, the degree to which a nurse feels part of a nursing team, the quality of the guidance at work from other nurses, and relationships with multi-professional staff; the factor of work conditions and reward, related to satisfaction from the workload (number of staff members), the scope of duties and the degree to which salary fits efforts at work and the factor managerial support, which reflected the satisfaction with the relationship with the head nurse.

The main outcome variable, willingness to stay in the nursing profession for the next 5 years, was measured on a dichotomous scale: (yes; no).

### Data analysis

We used t-tests to investigate the associations of each component separately with the intention of staying in the profession. The association between intention to stay and demographic and occupational characteristics was assessed with chi-square tests. Multivariable analysis with a logistic regression approach with willingness to stay in the profession (as a dependent variable) was used, while adjusted for variables found significantly associated with the outcome in univariate analyses. Before incorporating independent variables into the multivariable analysis, we evaluated the correlation between variables by employing Kendall’s Tau coefficient. For all analyses performed, *p* < 0.05 was considered statistically significant. Analyses were carried out using SPSS v.25.0 (IBM, US).

## Results

Demographic and employment characteristics of the study participants (n = 216) are shown in Table 1. Over half of the newly licensed nurses (53.7%) secured their initial employment within 1 week of receiving the government licensing examination results. A significant majority (63.4%) confirmed that they were employed in their preferred workplace. Most novice nurses began their careers in a hospital setting (82.6%), while others commenced their professional journey in community-based workplaces. A notable percentage of these new nurses (89.8%) expressed their intention to remain in the profession for the next 5 years.

**Table 1:** Willingness to stay in the profession by demographic and professional characteristics, univariate analysis

Demographic and professional characteristics	Professional Plans			Total n = 216
	Reported willingness to stay in the profession n = 194	Reported willingness to leave the profession n = 22	*p*	
Age (years) mean (SD)	27.8 (3.2)	28.6 (3.6)	.226	27.8 (3.3)
Sex (%)				
Female	88.1	86.4	.734	88.0
Male	11.9	13.6		12.0
Family status (%)				
Married or living with a partner	67.5	63.6	.811	67.1
Other (single, divorced)	32.5	36.4		32.9
Parental status (%)				
Has children	36.6	40.9	.816	37.0
Does not have children	63.4	59.1		63.0
Birth country (%)				
Israel	76.3	77.3	.9	76.4
Other (93% are immigrants from Former Soviet Union)	23.7	22.7		23.6
Population group (%)				
Jewish	97.9	100	1.0	98.1
Arab	2.1	0		1.9
Level of religiosity (%)				
Secular	53.1	50.0	.296	52.8
Traditional	18.8	31.8		20.1
Religious	28.1	18.2		27.1
Place of work (%)				
Hospital	82.2	86.4	.773	82.6
Community	17.8	13.6		17.4
Form of employment (%)				
Full time job	44.8	68.2	**.032**	47.2
Part time job	55.2	31.8		52.8
Work-family conflict (%)				
Never or sometimes	71.6	50.0	**.036**	69.4
Always	28.4	50.0		30.6
Level of control over the work schedule (%)				
Low	4.6	27.3	**.002**	6.9
Medium/high	95.4	72.7		93.1
Advanced professional course (%)				
Willing to/taking an advanced professional course	96.9	77.3	**.002**	94.9
No plan of taking an advanced professional course	3.1	22.7		5.1
Continuation of academic education to MA degree (%)				
Willing to start/started MA degree	79.9	40.9	** < .0001**	75.9
No plan of studying for MA degree	20.1	59.1		24.1

The statistically significant finding appears in bold (*p* < 0.05)

### Comparative analysis

As shown in Table 1, no significant differences in the distribution of most demographic and occupational characteristics were found between nurses who reported willingness to leave the profession and those who reported willingness to stay. Among those intending to leave, these factors had higher representation than among those who intend to stay: working full time (68.2% vs. 44.8%, *p* = .032), reporting frequency of work disturbances to everyday life as “always” (50.0% vs. 28.4%, *p* = .036), having low control over their work schedule (27.3% vs. 4.6%, *p* = .002), planning not to continue to the advanced course (22.7% vs. 3.1%, *p* = .002) and planning not to continue their academic education (59.1% vs. 20.1%,*p* < .0001).

### Regression analysis

**Fig. 2:**
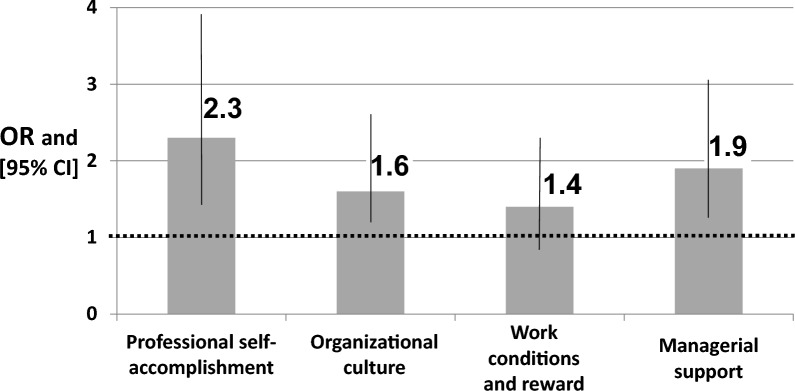
The intention to stay in the profession: Multivariable Logistic regression model. Multivariable logistic analysis with the intention to stay in the profession as a dependent variable and four components of job satisfaction as an independent variable, adjusted for form of employment, level of control over the work schedule, frequency of work disturbances to everyday life, and willingness to continue education (professional and academic)

Form of employment, level of control over the work schedule, frequency of work disturbances to everyday life, willingness to continue education (professional and academic), and four components of job satisfaction were included in a multivariable analysis with intention to stay in the profession as a dependent variable (Fig. 2). The model explained 38.0% of the variance in the intention to stay in the profession.

*Professional self-accomplishment* was significantly and positively associated with intention to stay in the profession; elevation of one standard deviation of this component was associated with more than a two-fold increase in the odds for staying in the profession (OR = 2.3, 95% CI 1.3–3.9). In simpler terms, higher professional self-accomplishment is strongly linked to a greater desire to stay in the profession and an increase in self-accomplishment makes it more than twice as likely that someone will want to stay in their profession. This component contributed 10% to the variance in intention to stay.

Independently, managerial support also contributed 10% to the variance in intention to stay in the profession. This factor was significantly associated with willingness to stay (OR = 1.9, 95% CI 1.2–3.0).

Organizational culture was significantly associated with the intention to stay in the profession, with an almost two-fold increase in odds of staying in the profession (OR = 1.6; 95% CI 1.1–2.5). This component contributed 4% to the variance in willingness to stay.

Work conditions and reward were not significantly associated with the odds of staying in the profession. This component contributed only 2% to the variance in intention to stay in the profession.

## Discussion

Current research examined the relationships between individuals and employment characteristics, job satisfaction, and willingness to stay in the profession among novice nurses. Among the components of job satisfaction, professional self-accomplishment was found to be the most important component in explaining professional satisfaction and strongly contributed to the intention to stay in the profession. This component represents the perception of a diverse and challenging job, a sense of accomplishment and meaningful contribution to patient care, as well as the prospect of career progression and autonomy in the job. A sense of achievement is vital for creating a productive and healthy work environment. A qualitative study among hospital nurses revealed that a sense of mission, accomplishment, passion, and the meaningful nature of nursing form a ‘positive energy’—a lasting inner strength not easily influenced by external factors. This positive energy stimulates retention of staff nurses [42]. In a longitudinal study among registered nurses, developmental opportunities significantly increased the sense of meaningfulness at work, leading to a notable decrease in both burnout and the intention to leave the nursing profession [29]. A job that offers continuous opportunities for professional development by fostering the acquisition of new knowledge and skills, contributes to enhanced retention. Hospitals with detailed transition programs for newly licensed nurses exhibited higher retention rates, with employed nurses reporting lower stress levels and increased job satisfaction [43]. Importantly, following the formal program, these new nurses were encouraged to participate in various system activities, including committees, unit projects, grand rounds, and other institutional career development and learning opportunities [43].

Another pivotal factor impacting the intention to remain in the nursing profession is the organizational culture. Its importance might be explained by the uniquely vulnerable position of novice nurses in the collective. During the first months of work, new nurses experience a transition from a supportive student environment to an open workforce. Lack of a sense of social belonging, insufficient skill-set confidence, and limited nursing experience among novice nurses make the first period of employment challenging [12, 13, 15, 44, 45]. Additionally, anxieties related to communication with physicians, patients, and senior nurses, role expectations, and unsupportive environments contribute to the challenges faced by novice nurses. Without sufficient relief from these stressors, achieving a successful transition becomes challenging, leading to emotional exhaustion and motivating new nurses to exit the profession [45]. Lack of support or even hostile behavior toward novice nurses can be detrimental. Approximately 17.5% of new nurses leave their first job within a year [46], and 60% of those quitting their job within the first 6 months do so due to bullying from co-workers [47]. According to previous studies, the frequency of workplace bullying reported by novice nurses during their first year of work is around 30% [48].

The organizational culture represents co-workers’ relationships in the collective, the feeling of belonging to the group, and the possibility of learning from more senior co-workers. The degree of unity within the nursing team was found to be associated with greater occupational satisfaction [49] and higher retention [50]. The possibility of learning and getting guidance from senior colleagues is crucial for young nurses, as it has been found that novice nurses intending to leave the profession reported a lack of support and feelings of isolation [51]. An inclusive and supportive workplace environment and colleague encouragement serve as a valuable resource for novice nurses. When experienced, these elements can alleviate stress and improve patient care by creating learning opportunities and providing observational role models, verbal support, and encouragement [50]. During the time of the pandemic, co-workers` support became even more important. Work team identification buffered against stress and burnout during COVID-19. Higher team identification was associated with significantly less work stress and burnout [52] and better mental well-being during the pandemic [4].

The relationship with a multi-professional team is another element of organizational culture. Collaboration among healthcare providers fosters continuous improvement in decision-making processes, enhancing patient outcomes [53, 54]. Improved teamwork and communication are the most important factors for providing safe and high-quality care and for the job satisfaction of healthcare workers [55, 56]. In particular, previous studies have underlined the importance of nurse-physician collaboration as this ensures shared goals and reciprocal duties to provide high-quality care to resolve patient problems [57, 58]. The lack of satisfactory professional relationships between the physicians and the nurses leads to burnout and stress among nurses [58–60].

Finally, the factor of managerial support was significantly associated with the intention to stay in the profession. This finding is consistent with the evidence that the transformational leadership style of nursing managers is associated with higher retention [61–63]. The four components of transformational leadership are inspirational motivation, idealized influence, intellectual stimulation, and individualized consideration. Transformational leadership style is characterized by the individual attention that nursing managers demonstrate towards subordinates by acknowledging their own strengths and weaknesses. Managerial support and good communication with supervisors were consistently associated with nurses’ intentions to stay in the profession [64, 65]. Supervisory support factors hold the most weight in relation to the turnover of nurses [66]. One of the important assignments of the head nurse is to manage the work scheduling process [67]. Appropriate work scheduling is consistently found to be associated with higher professional satisfaction, lower burnout, and greater intentions to stay. Having a say in scheduling allows nurses to manage the demands of work and home[67, 68]. Such conflicts result in lower job satisfaction [69–71] and more intentions to quit [72]. In this study, greater control over the work schedule was significantly associated with the intention to stay in the profession in the univariate analysis. However, as nurse managers played a crucial role in managing scheduling, after adjustment for the component of organizational culture (this component included assessment of nurse for “the amount of support and consideration of my needs I receive from my supervisor”) the association between control over the work schedule and intention to stay in the profession was no longer significant.

Work conditions and rewards were not significantly associated with the intention for retention. This finding was different from the conclusion of the review of nine studies, which found that poor working conditions contribute to a motivation to leave the nursing profession [73].

Discrepancies in findings may stem from differences in the study populations, with this study specifically including only novice nurses. It was previously found that younger nurses placed greater value on mentally challenging work and exciting work than their older colleagues [74].

### Study strength

The study encompassed the entire population of novice nurses/graduates rather than relying on a sample. The exceptionally high response rate mitigated the potential for selection bias.

Our study provides new insights, and we believe these contributions meaningfully advance the understanding of factors that promote retention among novice nurses and provide specific interventions to support workforce sustainability in nursing.

Our study specifically examines predictors of intention to stay and focuses on novice nurses within the first years of their careers. This early-career focus is critical in addressing the high turnover rates among this population.

The current study aimed to identify key predictors (professional self-accomplishment, managerial support, and organizational culture as key factors associated with the intention to stay in the profession). While these factors have been studied in general nursing populations, our study quantifies their impact specifically for novice nurses, offering a nuanced understanding of what supports retention at this stage.

The study’s findings form a foundation for evidence-based practical recommendations and actionable strategies for healthcare managers aimed at improving novice nurse retention, such as fostering environments that enhance professional self-accomplishment and providing targeted managerial support. These recommendations offer practical guidance for addressing novice nurses’ unique challenges during their transition to practice.

### Study limitations

This study assessed intentions to leave the profession, while the actual probability of leaving remains unclear. Nonetheless, intention to leave the current job or the profession was reported as the most accurate predictor of future actual turnover [34, 75]. Follow-up of these novice nurses (our graduates) will shed light on actual retention or leaving the profession during the coming years.

The cross-sectional study design does not allow a causal inference about predicting the tendency to stay in the nursing profession.

The regression model explained 38% of the variance in the intention to stay in the profession. While this is not a high percentage, it aligns with expectations in behavioral research, where a substantial portion of variance often remains unexplained due to the complexity of human behavior.

One of the key limitations of this study is that the data were collected from a single academic institution, which may limit the generalizability of the findings to other institutions. However, it is important to note that the nursing curriculum in Israel is largely regulated by the Ministry of Health, which sets the core courses and competencies required for all accredited nursing programs. This means that, despite being conducted at only one institution, the study’s findings are based on a curriculum that is consistent across institutions, ensuring that many aspects of the educational experience are standardized. Nevertheless, the findings may not fully reflect the diversity of nursing programs at other institutions that could differ in teaching methods, faculty experience, or student demographics. Future research could expand the scope by including multiple institutions to further examine how such variables may influence the outcomes. Additionally, our graduate population comprises diverse subgroups in terms of gender, ethnicity, and level of religiosity. This includes both Jewish and Arab students, as well as students representing a wide spectrum of religious observance and marital statuses. The study population reflects the proportion of male graduates in nursing schools across Israel. However, determining the extent to which this population represents the broader nursing student demographic remains challenging, as detailed characteristics, beyond the proportion of males, are not well-documented.

Finally, another limitation of this study is the relatively small number of novice nurses (n = 22) who reported an intention to leave the profession, which may affect the generalizability of the findings. However, it is worth noting that the results are consistent with existing literature on nurses in general, supporting the validity of the conclusions drawn.

### Policy implications and recommendations

This study revealed that professional self-accomplishment and managerial support were significantly and positively associated with the intention to stay in the profession. To enhance the retention of novice nurses, managers, such as head nurses, may implement the following strategies specifically focused on the successful integration of novice nurses:

- Incorporating customized mentorship programs, including tailored mentorship programs for novice nurses based on their specific needs (e.g., emotional, technical, or career advancement). This could foster a more personalized integration experience, enhancing retention.
- Fostering interprofessional collaboration opportunities throughout the implementation of structured interprofessional teamwork initiatives, such as joint learning and problem-solving sessions with physicians, therapists, and other healthcare professionals. These sessions can build stronger professional networks, enhancing engagement and reducing feelings of isolation.
- Introducing flexible career pathways and career progression opportunities to novice nurses can play a critical role in enhancing job satisfaction and retention. By offering them the chance to explore diverse specialties early in their careers, this approach addresses a common source of dissatisfaction—limited growth opportunities. This flexibility not only empowers nurses to tailor their career trajectories based on their evolving interests and strengths, but it also helps them envision long-term possibilities within the profession. Providing such varied experiences early on can foster greater engagement, commitment, and professional development, which are key factors in retaining novice nurses.
- Implementing psychological well-being interventions aimed at strengthening the psychological resilience of novice nurses is essential for supporting their long-term retention. Programs such as stress management workshops and peer support groups, designed specifically for nurses in the early stages of their careers, can provide vital preventive measures against burnout. These initiatives offer a safe space for novice nurses to share their experiences, manage the challenges of transitioning into their new roles, and develop coping strategies. By fostering emotional well-being and resilience, these programs not only reduce stress but also promote a healthier, more supportive work environment, ultimately enhancing job satisfaction and professional longevity.
- Creating leadership pathways and early leadership development programs aimed at novice nurses, providing them with the skills and confidence to take on leadership roles in their units, which can increase job satisfaction and long-term commitment.
- Increasing recognition through the implementation of peer-nominated awards can significantly enhance the sense of community and appreciation within the workplace. These awards allow colleagues to recognize and celebrate each other’s contributions, fostering a supportive and inclusive environment. By empowering peers to acknowledge outstanding efforts and achievements, this initiative can boost morale, strengthen team cohesion, and reinforce a culture of mutual respect. Such recognition is particularly impactful for novice nurses, as it validates their hard work and encourages continued dedication to the profession, thereby contributing to improved job satisfaction and retention.

## Conclusions

Self-accomplishment, healthier organizational culture, and better managerial support were significantly associated with novice nurses’ intention to stay in the profession. This study underscores the important role of professional self-accomplishment and managerial support in enhancing retention among novice nurses. To enhance retention, managers can implement strategies such as tailored mentorship programs, promoting interprofessional collaboration, offering flexible career pathways, implementing psychological resilience initiatives, fostering early leadership development, and introducing peer recognition programs. These measures address key challenges while fostering job satisfaction, engagement, and long-term commitment.

## Data Availability

The data supporting this study’s findings are available from the corresponding author, upon reasonable request. The data are not publicly available due to the decision of the Ethical Committee.
